# Fracture Healing in Elderly Mice and the Effect of an Additional Severe Blood Loss: A Radiographic and Biomechanical Murine Study

**DOI:** 10.3390/bioengineering10010070

**Published:** 2023-01-05

**Authors:** Katrin Bundkirchen, Weikang Ye, Aleksander J. Nowak, Stefan Lienenklaus, Bastian Welke, Borna Relja, Claudia Neunaber

**Affiliations:** 1 Hannover Medical School, Department of Trauma Surgery, 30625 Hannover, Germany; 2 Experimental Radiology, Department of Radiology and Nuclear Medicine, Otto Von Guericke University, 39120 Magdeburg, Germany; 3 Hannover Medical School, Institute of Laboratory Animal Science, 30625 Hannover, Germany; 4 Hannover Medical School, Department of Orthopaedic Surgery, Laboratory for Biomechanics and Biomaterials, 30625 Hannover, Germany

**Keywords:** ageing, bone regeneration, hemorrhagic shock

## Abstract

Femoral fractures and severe bleeding frequently occur in old patients showing a delayed healing. As there are no studies investigating the combined effect of high age and severe blood loss on fracture healing, this was examined radiographically and biomechanically in this study. Therefore, young and old male mice were randomly assigned to three operation groups. In the fracture group (Fx), external fixator and osteotomy were applied to the femur. The combined trauma group (THFx) additionally received a pressure-controlled hemorrhage. Sham animals were only implanted with arterial catheter and external fixator. Sacrifice was performed after three weeks and bone healing was evaluated radiologically via µCT, as well as biomechanically using a three-point bending test. A decreased share of callus/total bone volume was observed in old mice with blood loss compared to old Fx. Hemorrhagic shock also reduced the trabecular number in old mice compared to Fx and young THFx. Moreover, a lower elastic limit in old Sham mice without fracture was revealed. Fracture combined with a high loss of blood further reduced the elastic limit in old mice compared to isolated Fx in old animals. In conclusion, this study showed that severe blood loss has a higher negative effect in old mice compared to young ones.

## 1. Introduction

Ageing is one of the 21st century’s most critical socioeconomic and medical challenges, impacting all sectors of society [1]. As the geriatric population increases, a better understanding of how bone healing changes with age is crucial for developing effective treatment strategies and providing better medical care for these patients. The risk of non-unions increases with age, and osteoporotic fractures are associated with higher morbidity and mortality rates and increased costs for the health care system [2,3,4,5]. This has been proven in different in vivo animal models, and it was shown that cartilage and bone formation, resorption, bone microporosity, bone marrow stem cell function and quantity, as well as vascular perfusion of femoral blood flow was altered in old animals [6,7,8,9,10,11,12]. Moreover, in the setting of orthopedic trauma, a severe blood loss, frequently following long bone fractures, is associated with high morbidity and mortality rates [13]. The biological process of bone regeneration fails due to several reasons showing impaired recovery like delayed healing or even development of non-unions caused by fibrocartilage persisting in the fracture gap for longer than six to nine months and non-bridging of the callus across the fracture [14,15,16]. Although experimental models of hemorrhagic shock were established to explore the underlying pathophysiological mechanisms, as well as hemorrhage-related immunological changes and to determine the effectiveness of various therapeutic strategies, the effects of trauma hemorrhage on bone healing are still not yet examined in detail [13,17,18,19,20,21,22,23,24].

As elderly people are more susceptible to hemorrhagic shock and advanced age is one of the main predictors of poor clinical outcome after trauma-induced hemorrhagic shock, more experimental research is needed to gain more knowledge concerning this topic [25,26]. Currently, there are no studies addressing the effect of high age on the bone regeneration process after fracture in combination with a severe blood loss. Therefore, this study was designed to analyze in a murine animal model the effect of age in young but fully grown adults in comparison to the old population on the bone healing capacity after an additional severe blood loss. Our hypotheses were that (1) high age has a negative effect on fracture healing compared to young adult mice suffering from an isolated fracture, and (2) an additional hemorrhagic shock has a strong influence on fracture healing in old and young mice.

## 2. Materials and Methods

### 2.1. Animal Husbandry

All animal experiments were carried out according to the German Animal Welfare Legislation, approved by the local institutional animal care and research advisory committee and permitted by the local government of Lower Saxony, Germany (approval number: 33.12-42502-04-17/2491). The ARRIVE guidelines for reporting animal research were implemented. A total of 42 male C57BL/6J mice (Janvier Labs, Le Genest-Saint-Isle, France) were used for the experiments. The animals were housed in individual cages and kept under standardized conditions in the Central Animal Laboratory of the Hannover Medical School.

### 2.2. Age Calculation

The human age groups of 18–25 years as young adults and 60–70 years as old were converted into corresponding murine age groups after extensive literature research. The publication of Dutta et al., in which the lifespan of a mouse was converted into human years, and the data of Geifman and Rubin et al., including various pathologies such as fractures, were consulted for calculation of the corresponding murine age for this study [13,14]. It can be summarized that a 70-year-old patient was reflected by a mouse age of 637 days (9.1 days per human year) in the paper from Dutta et al. and by 386 days (5.5 days per human year) in the database of Geifman et al. with the pathology “fracture” [13,14]. This results in an average of 7.3 days in the mouse, which is equivalent to one human year. Therefore, it was decided that mice reflecting young adult patients (18–25 years) should have a murine age between 17 and 26 weeks and old patients (60–70 years) should have a murine age between 64 and 72 weeks.

### 2.3. Operation Steps

Young and old mice were randomly assigned to one of three operation groups. Weight, activity, and lameness scores were recorded (see Supplementary Tables S1 and S2). All surgical procedures were conducted under deep inhalation anesthesia with isoflurane [27,28]. An intraoperative analgesia was performed by subcutaneous injection of carprofen (5 mg/kg body weight) and butorphanol (1 mg/kg body weight) in combination with local anesthesia of prilocaine hydrochloride in the operation areas. In group Fx, an external fixator with 100% rigidity (MouseExFix simple L 100%, RISystem, Davos, Switzerland) was applied to the right femoral bone, and a diaphyseal osteotomy was performed centrally between the two middle pins using a wire saw with a diameter of 0.44 mm (Gigly wire saw, RISystem, Davos, Switzerland). The animals of the combined trauma group (THFx) additionally received a pressure-controlled trauma hemorrhage. The left femoral artery was catheterized with polyethylene tubing (Becton Dickinson, Sparks, MD, USA), and the initial arterial blood pressure was measured using a blood measuring cell (FMI TBD-1222, Föhr Medical Instruments, Seeheim, Germany) connected to a blood pressure analyzer (Micro-Med, Louisville, KY, USA). The mean measured initial arterial blood pressure was 79.14 ± 8.15 mmHg in young and 84.20 ± 4.92 mmHg in old THFx mice. The blood measuring cell was then temporarily disconnected from the catheter for blood sampling. Within 30 min, as much blood was drawn to drop the mean arterial blood pressure to a target value of 35 ± 5 mmHg (see Supplementary Figure S1). The shock state of hypovolemia was maintained for another 60 min, resulting in a total shock phase of 90 min. Afterwards, four times the amount of blood taken, with a maximum of 2.4 mL, was reperfused via the catheter with bodywarm Ringer’s solution over a period of 30 min. The Sham animals only received implantation of the arterial catheter and the external fixator, but no blood loss or osteotomy was performed. All animals were allowed to move freely and fully load the operated leg. For continuation of analgesia, the animals received metamizole (200 mg/kg body weight) in the drinking water for three days and carprofen and butorphanol subcutaneously according to indication.

### 2.4. Harvesting Procedure

For both ex vivo µCT and biomechanical analyses, a stable bridged bone was needed because the external fixator had to be removed before evaluations. It was shown before that stable bridging of fractured bones can be achieved in young and healthy mice two weeks after surgery [8,9]. Therefore, the end point of three weeks for ex vivo analyses was chosen in this study as old animals might show a delayed healing. Anesthesia was performed by intraperitoneal injection of ketamine (75 mg/kg body weight) and medetomidine (1 mg/kg body weight). Killing was achieved by cardiac exsanguination and final cervical dislocation. Afterwards, the femoral bones were explanted and soft tissue, as well as muscles, and the external fixator were removed. Samples were stored at −20°.

### 2.5. µCT Analysis

Bones were evaluated via Siemens Inveon µCT (Siemens AG, Munich, Germany). In vivo scans were conducted under isoflurane anesthesia two weeks after surgery to save animal lives, as prior experiments showed significant differences between Fx and THFx after two weeks in young and healthy animals obtained via µCT [9]. Measurements were performed with a 2 mm AL filter, 80 kV, 500 µA and 360 projections with 150 msec integration each (magnification Med-High, binning 4) within 5 min. The “effective pixel size” was 53 µm-resulting in a calculated dose of ~30cGy. For ex vivo evaluation after three weeks, scans are performed with the following settings: 360°, 360 steps, 7 s integration/position with 55 kV, 500 µA, filter 1 (0.8 carbon), high magnification, and binning 1 resulting in 8.17µm effective pixel size. Afterwards, bones were restored at −20 °C. Both in vivo and ex vivo scans were analyzed by the program Inveon Research Workplace 4.2 to examine total bone volume—including cortical bone—and callus volume in the fracture area between the two middle pins with a field of interest of 1.2 mm height. The ex vivo scans were additionally used to examine trabecular thickness, number, and spacing in the callus area. The share of callus per total bone volume was calculated by dividing the volume of callus by the total bone volume in the fracture area.

### 2.6. Three-Point Bending Assay

Prior to the three-point-bending test, bones were thawed for two hours at room temperature and transferred into Ringer’s solution. As described previously, the three-point bending test was performed using a material testing machine (MTS MiniBionix I, Model 858, Eden Prairie, MN, USA) [29]. The maximum bending moment [Nmm], the stiffness [N/mm], and the elastic limit [N] were determined from the load-deformation-curves [20]. The maximum bending moment [Nmm] was calculated by multiplying the distance between the lower bearings [mm] with the maximum force [N] (highest point of the curve), divided by 4. Calculation of the stiffness [N/mm] was performed using an auxiliary line parallel to the slope of the linear part of the curve. The elastic limit representing the passage from elastic to plastic deformity was defined as the point at which the auxiliary line intersected the curve.

### 2.7. Statistical Evaluation

A power calculation for the biomechanical analysis revealed that six animals in each group should achieve 80% power at an alpha value of 0.05. The statistical data analysis was performed with the SPSS Statistics^®^ program version 26.0 (IBM, New York, NY, USA). First, the data were analyzed concerning normal distribution via Shapiro-Wilk test and by interpreting the distribution in the histogram. These evaluations revealed non-parametric data for all parameters. Outliers were determined by the program and excluded for associated parameter, respectively (see Supplementary Table S3). The Mann–Whitney U test was carried out for comparisons between two groups. The Kruskal–Wallis test for non-parametric data was used to compare all groups. The failure concerning multiple testing was taken into account using the Bonferroni correction. All results are represented as median value and 95% confidence interval with upper and lower bound. The statistical significance was set at *p* ≤ 0.05.

## 3. Results

### 3.1. Survival Rate, Physiological Response and Macroscopic Evaluation

**Figure 1:**
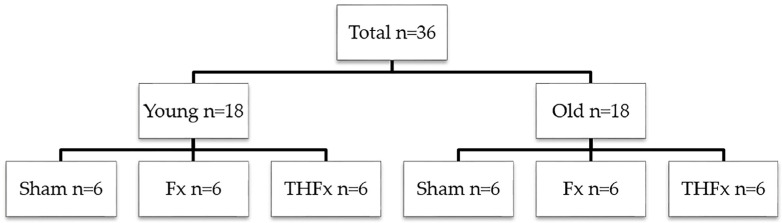
Flow chart of the experimental design. A total of 36 animals were included in the final statistical evaluation. Half of the animals were young (*n* = 18), and half old (*n* = 18). Randomization to one of the three operation groups was performed (*n* = 6 for Sham, Fx, THFx).

A total of 42 animals were operated for this experiment, but six animals (14.29%) were not included in the evaluation. Two animals did not survive until the observation endpoint: One old mouse died during the operation in reperfusion after trauma hemorrhage, and one young animal was found dead in the cage four days after THFx surgery for unknown reasons. Another animal could not be evaluated because of a huge cystic kidney and an extremely large bladder. One animal had to be excluded due to pin loosening. Two more mice were excluded because one fracture was not stable bridged, and the bone from the other mouse turned white and thick due to unknown reasons. Therefore, 36 animals were used for the respective analyses (*n* = 6 per group and age, flow chart see Figure 1).

Finally, young adult animals with an initial age of 17–21 weeks (average 18.4 ± 1.3 weeks) and old animals with 64–68 weeks (average 65.4 ± 1.6 weeks) were used. At the start of the experiment, the young mice weighed 29.0 ± 2.4 g and the old mice 32.9 ± 3.0 g. There were no significant differences in body weight, body temperature, or activity score of the groups. No animal showed a lameness of the fractured leg during the whole experiment. The average blood loss was 33.6 ± 2.8% (710.00 ± 58.65 µL) of the total blood volume in the young and 30.1 ± 4.4% (735.00 ± 96.76 µL) in the old mice. The total blood volume of a mouse is 70–80 mL/kg [30]. Therefore, the total blood volume was calculated in this study by multiplying the weight of the animals [g] with 75 µL. All 24 fractured bones were macroscopically stable before removal of the fixator.

### 3.2. µCT Evaluation In Vivo after Two Weeks

**Figure 2:**
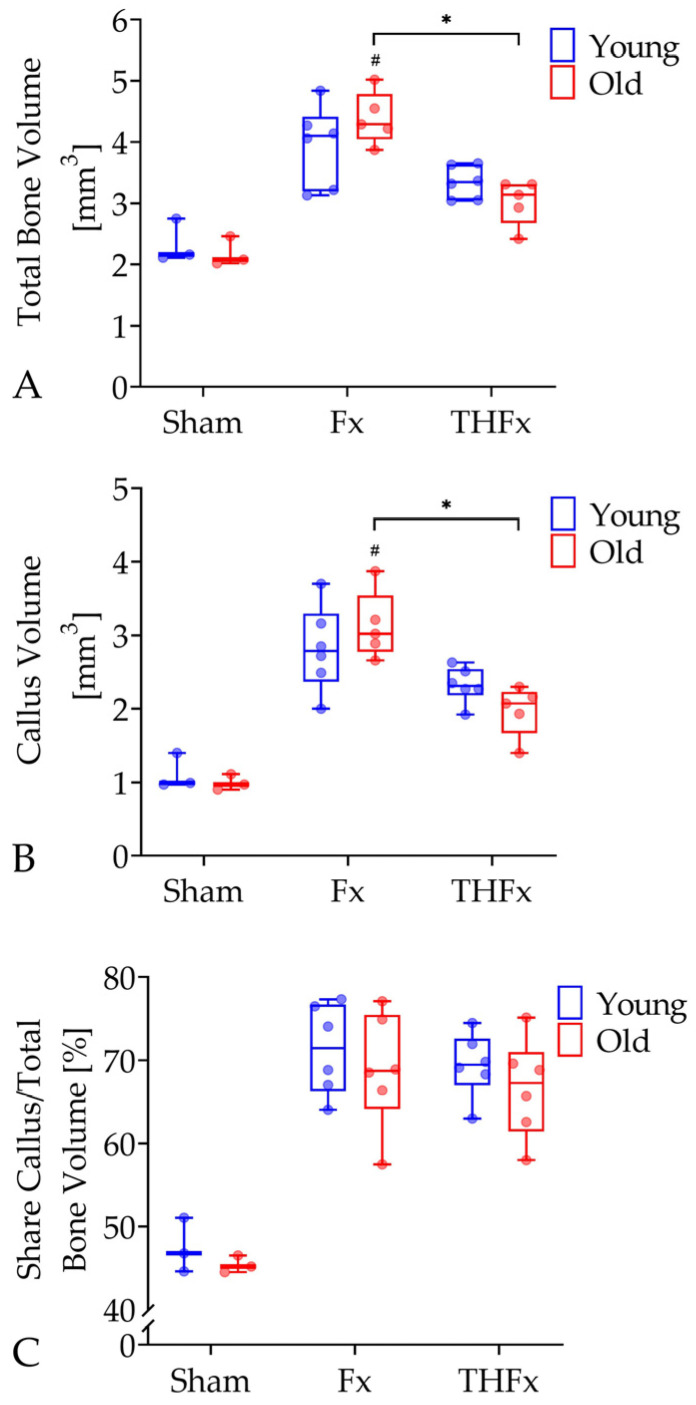
Analysis of the in vivo µCT parameters total bone volume (**A**), callus volume (**B**), and share callus/total bone volume (**C**) two weeks after the operation procedure. The Fx group showed significantly more total bone volume and callus volume than the THFx group and the Sham group in old mice. *n* = 3 for Sham and *n* = 5–6 for Fx and THFx. * *p* ≤ 0.05 vs. indicated group; # *p* ≤ 0.05 vs. associated Sham.

The analyzes of the in vivo µCT scans showed that in old mice a severe blood loss combined with a femoral fracture (THFx) led to a significant decrease of 26.81% in the parameter total bone volume with 3.14 mm^3^ (95% CI = 2.56–3.48 mm^3^) compared to an isolated fracture (Fx) with 4.29 mm^3^ (95% CI = 3.85–4.92 mm^3^; *p* = 0.008; Figure 2A). The same effect was also detectable in the young animals, but the decrease was only 18.54% and, therefore, not statistically significant. Apart from that, the Fx group showed significantly more total bone volume than the Sham group with 2.08 mm^3^ (95% CI = 1.59–2.79 mm^3^; *p* = 0.006) in old, but not in young mice. The old THFx group had a significant decrease of 31.46% in callus volume with 2.07 mm^3^ (95% CI = 1.54–2.79 mm^3^) in comparison to the old Fx group with 3.02 mm^3^ (95% CI = 2.56–3.70 mm^3^; *p* = 0.008; Figure 2B). This tendency was also found in young individuals with a not significant reduction of 17.20%. Furthermore, the callus volume in old mice was higher in the Fx group than in the Sham group with 0.97 mm^3^ (95% CI = 0.72–1.26 mm^3^; *p* = 0.006). This could not be seen in the young groups. No significant differences were found in the parameter share callus per total bone volume between all groups (Figure 2C).

### 3.3. µCT Evaluation Ex Vivo after Three Weeks

**Figure 3:**
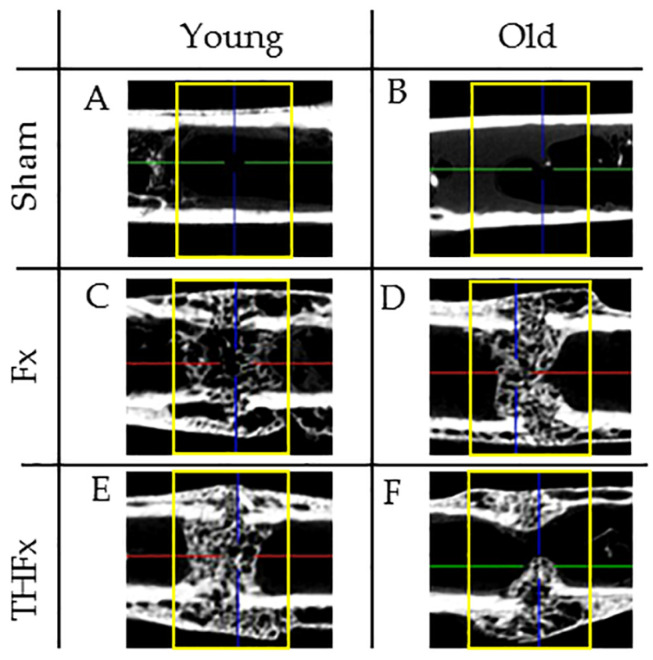
Representative sections of the ex vivo µCT scans three weeks after operation of the young (**A**,**C**,**E**) and old (**B**,**D**,**F**) Sham, (**A**,**B**) Fx (**C**,**D**), and THFx (**E**,**F**) groups. The region of interest (yellow rectangle in images) was the fracture position or corresponding area in the Sham groups for measuring bone parameters. Green, red and blue lines are orientation lines of the software displaying cutting levels in other planes (not shown).

Figure 3 shows six representative sections of the ex vivo µCT scans three weeks after the operation for young (A,C,E) and old (B,D,F) mice of the Sham (A,B) Fx (C,D), and THFx (E,F) groups.

**Figure 4:**
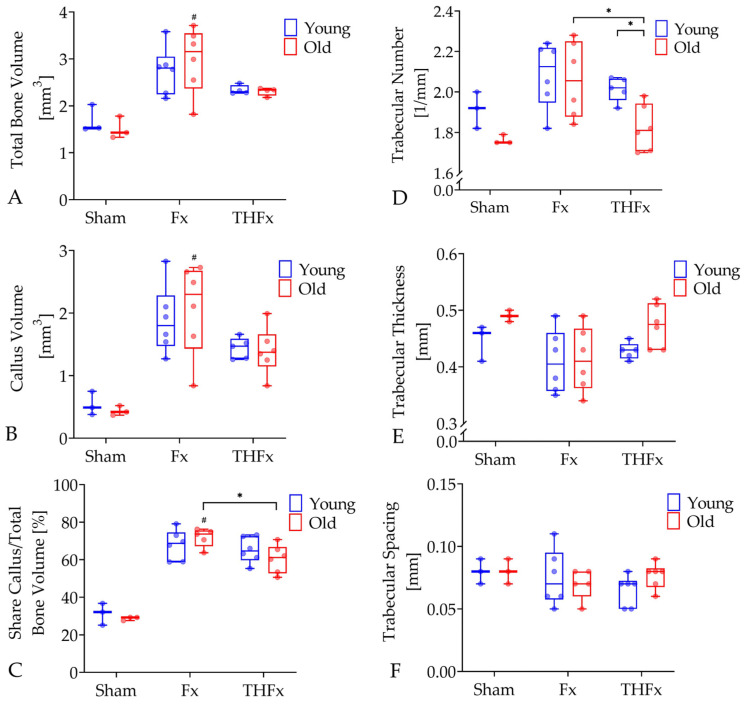
Analysis of the ex vivo µCT parameters total bone volume (**A**), callus volume (**B**), share callus/total bone volume (**C**), trabecular number (**D**), trabecular thickness (**E**) and trabecular spacing (**F**) three weeks after operation. For bone volume and callus volume, the Fx group indicated a significant difference compared with the Sham group in the elderly. In old mice, the share callus/total bone volume of the THFx group was significantly lower than in the Fx group in which values were higher than in Sham group. The trabecular number of the THFx group significantly decreased compared to the Fx group in old mice. For THFx group, young mice showed a significantly higher trabecular number than old mice. *n* = 3 for Sham and *n* = 5–6 for Fx and THFx. * *p* ≤ 0.05 vs. indicated group; # *p* ≤ 0.05 vs. associated Sham.

Old animals of the Fx group showed a significantly increased total bone volume with 3.16 mm^3^ (95% CI = 2.25–3.71 mm^3^) after an isolated fracture compared to old Sham mice without fracture with 1.43 mm^3^ (95% CI = 0.92–2.10 mm^3^; *p* = 0.023; Figure 4A). This effect occurred only in the old animals. Moreover, the callus volume of old animals was significantly higher in the Fx group with 2.3 mm^3^ (95% CI = 1.31–2.84 mm^3^) than in the Sham group with 0.42 mm^3^ (95% CI = 0.24–0.63 mm^3^; *p* = 0.022; Figure 4B) which did not appear in young mice. Apart from that, an additional trauma hemorrhage led to a significant decline of 16.94% in the share callus per total bone volume in old THFx animals with 61.18% (95% CI = 52.62–68.27%) in comparison to the old Fx mice with 73.66% (95% CI = 65.65%) without a severe blood loss (*p* = 0.030, Figure 4C). This difference was also observed in the young groups with a decrease of 5.94%, which was not statistically significant. The Fx groups also revealed significantly higher values for this parameter compared to the Sham group with 29.24% in old mice (95% CI = 26.26–31.13%; *p* = 0.014, Figure 4C) but not in young ones.

The evaluation of the trabecular structure in the callus revealed a significantly decreased trabecular number of 12.14% caused by blood loss in the THFx group with 1.81 mm^−1^ (95% CI = 1.70–2.03 mm^−1^) compared to the Fx group with 2.06 mm^−1^ (95% CI = 1.86–2.26 mm^−1^; *p* = 0.041; Figure 4D) in old individuals. This effect also occurred in the young mice with a reduction of 4.72%, but without significance. A high age moreover significantly reduced the trabecular number in old mice suffering from fractures in combination with trauma hemorrhage by 10.40% in contrast to the young ones with 2.02 mm^−1^ (95% CI = 1.94–2.08 mm^−1^; *p* = 0.017). A similar effect without significance of 2.83% less trabeculae was also visible in the Fx group.

For the parameters trabecular thickness and trabecular spacing, no significant differences were found between any of the groups (Figure 4E,F).

### 3.4. Biomechanical Evaluation after Three Weeks

**Figure 5:**
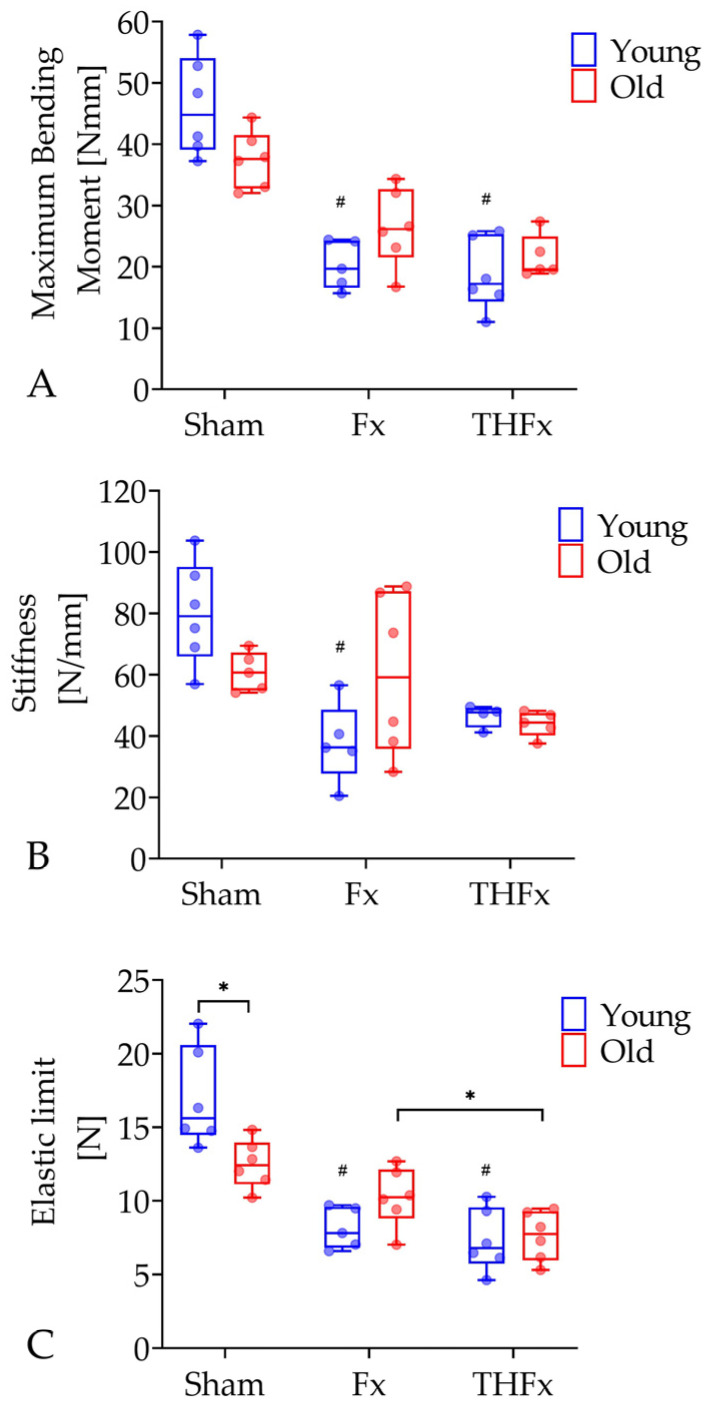
Evaluation of biomechanical parameters with maximum bending moment (**A**), stiffness (**B**), and elastic limit (**C**) three weeks after operation via three-point bending test. The maximum bending moment was significantly lower in bones of the Fx and THFx groups compared to the Sham group in young animals. In young mice, also the stiffness was significantly reduced in the Fx group compared to the Sham group. For old mice, the elastic limit significantly decreased in the THFx group compared to the Fx group. Old mice also demonstrated a lower elastic limit than young mice in the Sham group. Besides, the elastic limit in bones of Fx and THFx groups was significantly reduced compared to the Sham group in young mice. *n* = 4–6 for Sham, Fx and THFx. * *p* ≤ 0.05 vs. indicated group; # *p* ≤ 0.05 vs. associated Sham.

The results of the three-point bending test for the evaluation of the biomechanical parameter maximum bending moment, stiffness, and elastic limit are shown in Figure 5. The young animals of the THFx group with 17.20 Nmm (95% CI = 12.56–24.72 Nmm; *p* = 0.002), as well as of the Fx group with 19.68 Nmm (95% CI = 15.39–25.14 Nmm; *p* = 0.014) showed a significantly lower maximum bending moment compared to the young Sham mice with 44.8 Nmm (95% CI = 37.66–54.74 Nmm; Figure 5A). This observation was not visible in old mice.

For the parameter stiffness, the three-point bending test showed significantly lower values in the young Fx group with 36.21 N/mm (95% CI = 21.75–53.86 N/mm) compared to the young Sham group with 79.05 N/mm (95% CI = 62.45–97.59 N/mm; *p* = 0.006; Figure 5B). In the old animals, such an effect was not detectable.

The analysis of the parameter elastic limit revealed that a severe blood loss led to a significant reduction of 24.32% with 7.75 N (95% CI = 5.86–9.36 N) in the THFx group in comparison to an isolated fracture in the Fx group with 10.24 N (95% CI = 8.16–12.35 N; *p* = 0.022; Figure 5C) in old animals. A decrease of 13.06% was also detected in young mice but without statistical significance. Moreover, old mice of the Sham group demonstrated a significantly lower elastic limit with 12.42 N (95% CI = 10.77–14.22 N) than the young Sham mice with 15.63 N (95% CI = 13.44–20.48 N; *p* = 0.015). The parameter elastic limit also decreased significantly in young animals of the Fx group with 7.81 N (95% CI = 6.37–9.87 N; *p* = 0.026) and the THFx group with 6.79 N (95% CI = 5.10–9.52 N; *p* = 0.002) compared to the Sham group. This difference could not be observed in the old mice.

## 4. Discussion

Fractures of the long bones commonly occur in combination with a blood loss in the clinical situation [13,31,32]. Nowadays, a fracture of the femoral neck is assumed to result in a loss of 2 L of blood and a broken lower leg around 1 L. A blood loss of 0.75–1.25 L corresponds to a moderate hemorrhagic shock (Level II) while a severe trauma hemorrhage is reflected by a loss of 1.25–2 L (Level III) [33]. It is also known that particularly elderly patients are more sensitive to a loss of blood volume than young, healthy adults [33]. Therefore, the influence of trauma hemorrhage on fracture healing is of great importance for the large patient population of people over 60 years, who often break their thighs or other long tubular bones in the case of falls [34]. Thus, this study was designed to investigate age-related changes combining hemorrhagic shock with its influence on fracture healing in a murine model at an age of 17–21 weeks (young mice) and 64–68 weeks (old mice) reflecting 18–25-year-old young, fully grown adults and elderly people with an age of 60–70 years. The evaluation revealed that in old mice an additional hemorrhagic shock led to a significantly lower total bone and callus volume, a significantly decreased share of callus per total bone volume, significantly less trabecular structures and a significant reduction of the elastic limit. These effects did not occur in the young animals. Moreover, high age significantly reduced the elastic limit in unfractured bones and also the trabecular number after femoral fracture in combination with a severe blood loss.

Concerning the effect of hemorrhagic shock on fracture healing, our knowledge is still limited, and experimental in vivo animal studies in mostly young animals show controversial results, reaching from the often-postulated negative effects [13,17,24], that are also noticeable in the clinic, to no effect [23], and to even a tendency towards a positive effect of a blood loss on bone healing [18,21]. In a previous study, our working group analyzed the effect of a severe blood loss on fracture healing in young mice with an initial age of 12 weeks [19,20]. A delayed fracture healing after additional hemorrhagic shock in terms of a significantly higher share of callus per volume bone mass in the evaluation of the ex vivo mCT scans after two weeks was observed. The trauma hemorrhage also caused a significantly reduced maximum bending moment two weeks after fracture. In the study of Lichte et al., a pressure controlled hemorrhagic shock combined with a closed femoral fracture model with stabilization was performed in mice with an initial weight of 25 g [13]. Here, no significant differences in bone volume fraction between an isolated fracture and fracture combined with a severe blood loss measured by µCT at 21 days after fracture were found [13]. Moreover, there was no significant difference in the tensile strength after closed femoral fracture with or without pressure controlled hemorrhagic shock in mechanical testing [13]. These results are in line with the results of our studies for both not fully grown and adult young mice. In another study of Brady et al., a fixed-volume hemorrhagic shock rabbit model in combination with a femoral osteotomy was used [17]. Plain radiographs showed that a higher callus index occurred in the group with blood loss after the third postoperative week [17]. These findings are in line with our results for young animals but contrary to the decreased share of callus per total bone volume in old THFx mice after three weeks. In two other studies on rats, blood loss had a positive effect in terms of an enhanced systemic bone formation and improvement of both blood flow and the biomechanical outcome of bone healing [18,21]. Nevertheless, such positive effects of trauma hemorrhage may have been caused by differences in the study designs and are not in line with the findings of most other studies.

Concerning high age, numerous studies have shown that the repair of fractures is impaired in older animals. A study of Lopas et al. on mature young and old mice demonstrated that callus expansion and bone volume are significantly reduced with rising age [35]. Comparing 21-week-old with 107-week-old male C57BL/6 mice, they observed that the total volume, bone volume, bone volume/total callus volume, and trabecular thickness of young mice were significantly higher 15 and 20 days after fracture compared to the old mice group [35]. In contrast to that, our study revealed no significant reduction in the old mice with an isolated femoral fracture compared to the young ones for bone volume or trabecular thickness after 14 or 21 days. However, Lopas et al. used the age of 107 weeks for the old mice group, which is much older than 65-week-old mice as used in our study and might explain the different results. Borgiani et al. compared female C57Bl/6J adult mice (26-week-old) to elder mice (78-week-old), and ex vivo µCT analyses were performed at one, two, and three weeks after fracture [36]. They observed no significant differences in mineralized callus volume, total callus volume and mineralized callus volume fraction between young and old mice after three weeks [36]. These findings are comparable to our results. Further investigations in murine fracture models revealed that aging significantly affects the biomechanical properties and leads to a poor mechanical strength and delayed bone healing. Bak et al. compared young (3-month-old) and old (2-year-old) male rats with a closed fracture of the tibia and found that young animals had a higher stiffness, maximum load, and elastic modulus than old animals at day 80 after fracture [7]. Strube et al. using 12-week- and 12-month-old rats discovered that old rats had a lower stiffness and maximum torque at failure than young rats at 6 weeks after fracture [37]. These results were only partly reflected in our study as we only found a significant reduction in the elastic modulus.

As shown above, several in vivo animal experiments of bone healing concerning age or hemorrhagic shock were designed to predict human responses, but experimental in vivo studies addressing the effect of both high age and severe blood loss on fracture regeneration are still missing.

Although an established, standardized, reproducible mouse model was used in this study, there are several limitations that have to be considered. Bone healing was evaluated in only two age groups of mice. Apart from that, significant differences were only observable in the old groups. However, similar trends but smaller in the magnitude can be seen in the younger groups. The reason for this may be an insufficient study power of only *n* = 6 animals per group. Furthermore, just one selective final evaluation time point of three weeks was assessed. This time point was chosen as a stable bridging of the fracture gap was necessary for the radiological and biomechanical analyzes which for young mice probably reflects the early remodeling stage and for older mice the hard callus formation stage [3,38]. Therefore, earlier detection time points may reveal more detailed findings especially in molecular biology and immunogenetics, as well as on cellular level after histological evaluation. In order to gain insights into the healing process also after two weeks, but still save animal lives, in vivo µCT scans were performed additionally. In order to keep a possible negative effect of the radiation on the animals and the fracture healing as low as possible, only a short scan duration with low radiation was chosen for the in vivo recordings resulting in a lower resolution. Due to the surrounding tissue in the living animal and the influence on the data, the absolute values of the in vivo scans are, unfortunately, not comparable to the ex vivo scans. Moreover, here only male mice were used. This was done to avoid the influence of estrogen levels on the fracture healing due to the female sexual cycle. Therefore, another study comparing the effect of age and severe blood loss in both sexes should be conducted in the future.

In the future, based on the results generated in this study, histological evaluations shall be investigated two and three weeks after surgery concerning cartilage and bone tissue and the cells involved in the healing process. Moreover, potential biological mechanisms on the molecular level behind the observed differences will be deeply analyzed using micro arrays. These results will be further verified by qRT-PCR and Western Blot analyzes.

## 5. Conclusions

In conclusion, this study was the first analyzing the effect of high age on bone healing in a combinatory fracture model with a severe blood loss. The results showed that severe blood loss has little to no adverse effect on fracture healing in young mice, but appears to have a significant, adverse effect on fracture healing in older animals. Thus, the ability of bone to heal appears to be impaired by hemorrhagic shock with age. A deeper knowledge in this field could have beneficial effects on the treatment options and help to estimate the possible risks in older patients suffering from long bone fractures combined with blood loss.

## Data Availability

The data presented in this study are available on request from the corresponding author on reasonable request.

## Supplementary Materials

The following supporting information can be downloaded at: https://www.mdpi.com/article/10.3390/bioengineering10010070/s1, Figure S1: Initiation of trauma hemorrhage. Table S1: Activity score. Table S2: Lameness score. Table S3: Total animal numbers and valid number after exclusion of outliers.

## References

[1] Department of Economic and Social Affairs (2017). World Population Ageing 2017 Highlights.

[2] Borgstrom F., Karlsson L., Ortsater G., Norton N., Halbout P., Cooper C., Lorentzon M., McCloskey E.V., Harvey N.C., Javaid M.K. (2020). Fragility fractures in Europe: Burden, management and opportunities. Arch. Osteoporos..

[3] Clark D., Nakamura M., Miclau T., Marcucio R. (2017). Effects of Aging on Fracture Healing. Curr. Osteoporos. Rep..

[4] Gorter E.A., Reinders C.R., Krijnen P., Appelman-Dijkstra N.M., Schipper I.B. (2021). The effect of osteoporosis and its treatment on fracture healing a systematic review of animal and clinical studies. Bone Rep..

[5] Meinberg E.G., Clark D., Miclau K.R., Marcucio R., Miclau T. (2019). Fracture repair in the elderly: Clinical and experimental considerations. Injury.

[6] Aho A.J. (1966). Electron microscopic and histologic studies on fracture repair in old and young rats. Acta Chir. Scand. Suppl..

[7] Bak B., Andreassen T.T. (1989). The effect of aging on fracture healing in the rat. Calcif. Tissue Int..

[8] Hemmatian H., Laurent M.R., Bakker A.D., Vanderschueren D., Klein-Nulend J., van Lenthe G.H. (2018). Age-related changes in female mouse cortical bone microporosity. Bone.

[9] Histing T., Kuntz S., Stenger D., Scheuer C., Garcia P., Holstein J.H., Klein M., Pohlemann T., Menger M.D. (2013). Delayed fracture healing in aged senescence-accelerated P6 mice. J. Investig. Surg..

[10] Lu C., Miclau T., Hu D., Hansen E., Tsui K., Puttlitz C., Marcucio R.S. (2005). Cellular basis for age-related changes in fracture repair. J. Orthop. Res..

[11] Prisby R.D., Ramsey M.W., Behnke B.J., Dominguez J.M., Donato A.J., Allen M.R., Delp M.D. (2007). Aging reduces skeletal blood flow, endothelium-dependent vasodilation, and NO bioavailability in rats. J. Bone Miner. Res..

[12] Sethe S., Scutt A., Stolzing A. (2006). Aging of mesenchymal stem cells. Ageing Res. Rev..

[13] Lichte P., Kobbe P., Pfeifer R., Campbell G.C., Beckmann R., Tohidnezhad M., Bergmann C., Kadyrov M., Fischer H., Gluer C.C. (2015). Impaired Fracture Healing after Hemorrhagic Shock. Mediat. Inflamm..

[14] Claes L., Recknagel S., Ignatius A. (2012). Fracture healing under healthy and inflammatory conditions. Nat. Rev. Rheumatol..

[15] Marsell R., Einhorn T.A. (2010). Emerging bone healing therapies. J. Orthop. Trauma..

[16] Marsell R., Einhorn T.A. (2011). The biology of fracture healing. Injury.

[17] Brady J., Hardy B.M., Yoshino O., Buxton A., Quail A., Balogh Z.J. (2018). The effect of haemorrhagic shock and resuscitation on fracture healing in a rabbit model: An animal study. Bone Jt. J..

[18] Bumann M., Henke T., Gerngross H., Claes L., Augat P. (2003). Influence of haemorrhagic shock on fracture healing. Langenbecks Arch. Surg..

[19] Bundkirchen K., Macke C., Angrisani N., Schack L.M., Noack S., Fehr M., Krettek C., Neunaber C. (2018). Hemorrhagic shock alters fracture callus composition and activates the IL6 and RANKL/OPG pathway in mice. J. Trauma Acute Care Surg..

[20] Bundkirchen K., Macke C., Reifenrath J., Schack L.M., Noack S., Relja B., Naber P., Welke B., Fehr M., Krettek C. (2017). Severe Hemorrhagic Shock Leads to a Delayed Fracture Healing and Decreased Bone Callus Strength in a Mouse Model. Clin. Orthop. Relat. Res..

[21] Lucas T.S., Bab I.A., Lian J.B., Stein G.S., Jazrawi L., Majeska R.J., Attar-Namdar M., Einhorn T.A. (1997). Stimulation of systemic bone formation induced by experimental blood loss. Clin. Orthop. Relat. Res..

[22] Moochhala S., Wu J., Lu J. (2009). Hemorrhagic shock: An overview of animal models. Front. Biosci..

[23] Starr A.J., Welch R.D., Eastridge B.J., Pierce W., Zhang H. (2002). The effect of hemorrhagic shock in a caprine tibial fracture model. J. Orthop. Trauma.

[24] Wichmann M.W., Arnoczky S.P., DeMaso C.M., Ayala A., Chaudry I.H. (1996). Depressed osteoblast activity and increased osteocyte necrosis after closed bone fracture and hemorrhagic shock. J. Trauma.

[25] Hooper N., Armstrong T.J. (2022). Hemorrhagic Shock. StatPearls.

[26] Vanzant E.L., Hilton R.E., Lopez C.M., Zhang J., Ungaro R.F., Gentile L.F., Szpila B.E., Maier R.V., Cuschieri J., Bihorac A. (2015). Advanced age is associated with worsened outcomes and a unique genomic response in severely injured patients with hemorrhagic shock. Crit. Care.

[27] Dutta S., Sengupta P. (2016). Men and mice: Relating their ages. Life Sci..

[28] Geifman N., Rubin E. (2013). The mouse age phenome knowledgebase and disease-specific inter-species age mapping. PLoS ONE.

[29] Leppanen O., Sievanen H., Jokihaara J., Pajamaki I., Jarvinen T.L. (2006). Three-point bending of rat femur in the mediolateral direction: Introduction and validation of a novel biomechanical testing protocol. J. Bone Miner. Res..

[30] Dülsner A., Hack R., Krüger C., Pils M., Scherer K., Schmelting B., Schmidt M., Weinert H., Jourdan T. (2017). Fachinfomation aus dem Ausschuss für Tierschutzbeauftragte und dem Arbeitskreis 4 in der TVT. Empfehlung zur Blutentnahme bei Versuchstieren, Insbesondere Kleinen Versuchstieren.

[31] Lieurance R., Benjamin J.B., Rappaport W.D. (1992). Blood loss and transfusion in patients with isolated femur fractures. J. Orthop. Trauma.

[32] Wertheimer A., Olaussen A., Perera S., Liew S., Mitra B. (2018). Fractures of the femur and blood transfusions. Injury.

[33] Marx G., Muhl E., Zacharowski K., Zeuzem S. (2015). Die Intensivmedizin.

[34] Beerekamp M.S.H., de Muinck Keizer R.J.O., Schep N.W.L., Ubbink D.T., Panneman M.J.M., Goslings J.C. (2017). Epidemiology of extremity fractures in the Netherlands. Injury.

[35] Lopas L.A., Belkin N.S., Mutyaba P.L., Gray C.F., Hankenson K.D., Ahn J. (2014). Fractures in geriatric mice show decreased callus expansion and bone volume. Clin. Orthop. Relat. Res..

[36] Borgiani E., Figge C., Kruck B., Willie B.M., Duda G.N., Checa S. (2019). Age-Related Changes in the Mechanical Regulation of Bone Healing Are Explained by Altered Cellular Mechanoresponse. J. Bone Miner. Res..

[37] Strube P., Sentuerk U., Riha T., Kaspar K., Mueller M., Kasper G., Matziolis G., Duda G.N., Perka C. (2008). Influence of age and mechanical stability on bone defect healing: Age reverses mechanical effects. Bone.

[38] Jensen D.M. (2018). Diagnosis and treatment of definitive diverticular hemorrhage (DDH). Am. J. Gastroenterol..

