# Supplementary material for: Fracture Healing in Elderly Mice and the Effect of an Additional Severe Blood Loss: A Radiographic and Biomechanical Murine Study
Source: Bioengineering (Basel). 2023 Jan 5;10(1):70. doi: 10.3390/bioengineering10010070 (PMC9855159; doi:10.3390/bioengineering10010070)
Supplement: Supplementary file 1 [file bioengineering-10-00070-s001.zip › bioengineering-2071941-supplementary Figure S1.pdf]

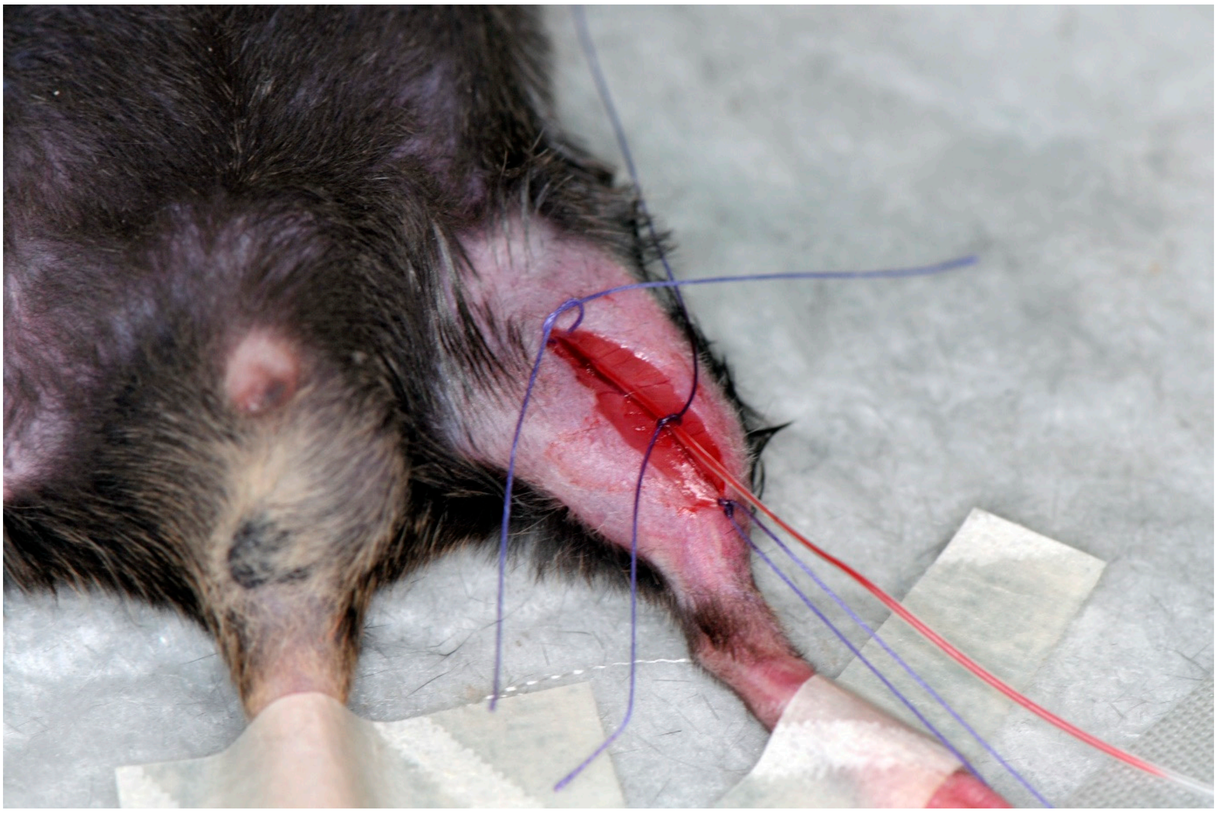

Supplementary Figure S1. Initiation of trauma hemorrhage. After insertion of a catheter into the left femoral artery, the mean arterial blood pressure was measured by a blood pressure analyzer, which was connected to a blood pressure measuring cell. Alternately, blood was collected via a syringe, after disconnecting the measuring devices, to maintain a mean arterial blood pressure of 35 ± 5 mmHg over 90 minutes. Afterwards, four times of the amount of taken blood was reinfused by pre-heated body-temperature Ringer's solution via the catheter within 30 minutes.
