# Supplementary material for: Fracture Healing in Elderly Mice and the Effect of an Additional Severe Blood Loss: A Radiographic and Biomechanical Murine Study
Source: Bioengineering (Basel). 2023 Jan 5;10(1):70. doi: 10.3390/bioengineering10010070 (PMC9855159; doi:10.3390/bioengineering10010070)
Supplement: Supplementary file 1 [file bioengineering-10-00070-s001.zip › bioengineering-2071941-supplementary Table S1.pdf]

Supplementary Table S1. Activity score. The activity of the animals was regularly monitored by assessing the behavior and general condition using an activity score.

Activity Score	Quality	Behaviour	General Condition
1	very active	lively, attentive, curious, species-typical movements	fur smooth, shiny; eyes clear, shiny; orifices clean
2	active	attentive, species-typical movements	fur smooth, shiny; eyes clear, shiny; orifices clean
3	reduced active	attentive, calm, reduced movement, species-typical posture	fur smooth, shiny; eyes clear, shiny; orifices clean
4	quiet	animal calm, frequent pausing, limited reactions to environmental stimuli, reduced personal hygiene	fur dull, possibly erect; eyes no longer fully open
5	lethargic	self-isolation; no significant activity	fur erect; eyes closed; orifices sticky or damp; hunched posture
6	moribund	no activity, no reaction to environmental stimuli	eyes closed; lateral position; shallow breathing; convulsions; animal cold
