# Supplementary material for: Fracture Healing in Elderly Mice and the Effect of an Additional Severe Blood Loss: A Radiographic and Biomechanical Murine Study
Source: Bioengineering (Basel). 2023 Jan 5;10(1):70. doi: 10.3390/bioengineering10010070 (PMC9855159; doi:10.3390/bioengineering10010070)
Supplement: Supplementary file 1 [file bioengineering-10-00070-s001.zip › bioengineering-2071941-supplementary Table S2.pdf]

Supplementary Table S2. Lameness score. With this score, the load on the fractured leg was checked regularly.

**Lameness Score**	**Quality**	**Characteristics**
A	full weight bearing	full weight bearing on fractured leg; no signs of lameness
B	lameness	not full weight bearing on fractured leg; intermittent relief or hesitation
C	no weight bearing	no weight bearing on fractured leg; complete, permanent relief
