# Supplementary material for: Fracture Healing in Elderly Mice and the Effect of an Additional Severe Blood Loss: A Radiographic and Biomechanical Murine Study
Source: Bioengineering (Basel). 2023 Jan 5;10(1):70. doi: 10.3390/bioengineering10010070 (PMC9855159; doi:10.3390/bioengineering10010070)
Supplement: Supplementary file 1 [file bioengineering-10-00070-s001.zip › bioengineering-2071941-supplementary Table S3.pdf]

Supplementary Table S3. Total animal numbers and valid number after exclusion of outliers. The table shows the total number of animals used for every parameter as well as the valid number after excluding the outliers defined by the statistical program SPSS.

**Parameter**	**Young Sham**		**Young Fx**		**Young THFx**		**Old Sham**		**Old Fx**		**Old THFx**	
	**To-tal**	**Va-lid**	**To-tal**	**Va-lid**	**To-tal**	**Va-lid**	**To-tal**	**Va-lid**	**To-tal**	**Va-lid**	**To-tal**	**Va-lid**
***In vivo* Total Bone Volume**	3	3	6	6	6	6	3	3	6	5	6	5
***In vivo* Callus Volume**	3	3	6	6	6	6	3	3	6	5	6	5
***In vivo* Share Callus / Total Bone Volume**	3	3	6	6	6	6	3	3	6	6	6	6
***Ex vivo* Total Bone Volume**	3	3	6	6	6	4	3	3	6	6	6	4
***Ex vivo* Callus Volume**	3	3	6	6	6	5	3	3	6	6	6	6
***Ex vivo* Share Callus / Total Bone Volume**	3	3	6	6	6	6	3	3	6	5	6	6
**Trabecular Number**	3	3	6	6	6	5	3	3	6	6	6	6
**Trabecular Spacing**	3	3	6	6	6	6	3	3	6	5	6	6
**Trabecular Thickness**	3	3	6	6	6	5	3	3	6	6	6	6
**Maximum Bending Moment**	6	6	6	5	6	6	6	6	6	6	6	5
**Stiffness**	6	6	6	5	6	4	6	5	6	6	6	5
**Elastic Limit**	6	6	6	5	6	6	6	6	6	6	6	6
